# Juxta-articular extraskeletal myxoid chondrosarcoma mistaken for a benign cyst presenting with multiple lung metastases

**DOI:** 10.1016/j.radcr.2023.10.075

**Published:** 2023-11-27

**Authors:** Dmitriy Starostin, Ibrahim Azam, Michael Paddock, Malee S. Fernando, Scott Evans MBChB, Nikhil Kotnis

**Affiliations:** a Clinical Radiology Department, Sheffield Teaching Hospitals NHS Trust, Sheffield, UK; b Department of Radiology, Stoke Mandeville Hospital, Buckinghamshire Healthcare NHS Trust, Buckinghamshire, UK; c Medical Imaging Department, Perth Children's Hospital, Perth, Western Australia, Australia; d Division of Pediatrics, University of Western Australia, Perth, Western Australia, Australia; e School of Medicine, The University of Notre Dame Australia, Perth, Western Australia, Australia; f Division of Clinical Medicine, School of Medicine and Population Health, University of Sheffield, Sheffield, UK; g Sheffield Teaching Hospitals NHS Trust, Sheffield, UK; h The Royal Orthopedic Hospital, Birmingham, UK; i University of Birmingham, Birmingham, UK; j MSK Radiology Department, Sheffield Teaching Hospitals NHS Trust, Sheffield, UK

**Keywords:** Extraskeletal myxoid chondrosarcoma, EMC, Sarcoma, Paralabral cyst

## Abstract

Extraskeletal myxoid chondrosarcoma (EMC) is a malignant cartilage neoplasm usually encountered in the proximal extremities. We report the case of a 58-year-old male who presented initially with a 3-month history of cough. Initial staging demonstrated a right upper lobe mass with bilateral pulmonary nodules and moderate tracer uptake in the right lung mass and right groin on positron emission tomography imaging. Endobronchial ultrasound biopsy confirmed a histological diagnosis of EMC for which the patient underwent right upper lobe wedge resection. Pelvic MRI revealed a peripherally enhancing juxta-articular lesion within the region of the right obturator externus bursa, which was thought initially to represent either a ganglion or paralabral cyst. However, ultrasound-guided biopsy yielded identical histology to the resected lung mass leading to the diagnosis of primary EMC in the right groin with pulmonary metastases. The patient underwent surgical excision of the right groin mass with no local recurrence on the surveillance computed tomography at 5, 12, and 18 months but eventual disease recurrence in the right groin and further progression of the pulmonary metastases at 29 months. We emphasize that the contrast enhancement pattern of EMC can mimic a benign cystic lesion, in particular, when in a juxta-articular location, which has the potential to mislead radiologists and delay diagnosis and definitive treatment.

## Introduction

We report the case of metastatic extraskeletal myxoid chondrosarcoma (EMC) at presentation. The primary lesion was eventually discovered to arise from the juxta-articular right hip, specifically within the region of the obturator externus bursa. The MRI signal characteristics and enhancement pattern of the lesion closely resembled a large ganglion in a location where benign juxta-articular cysts are commonly encountered. The case serves as a cautionary tale that contrast enhancement on MRI may not always reliably differentiate EMC from a benign juxta-articular cyst.

## Case report

**Fig. 1:**
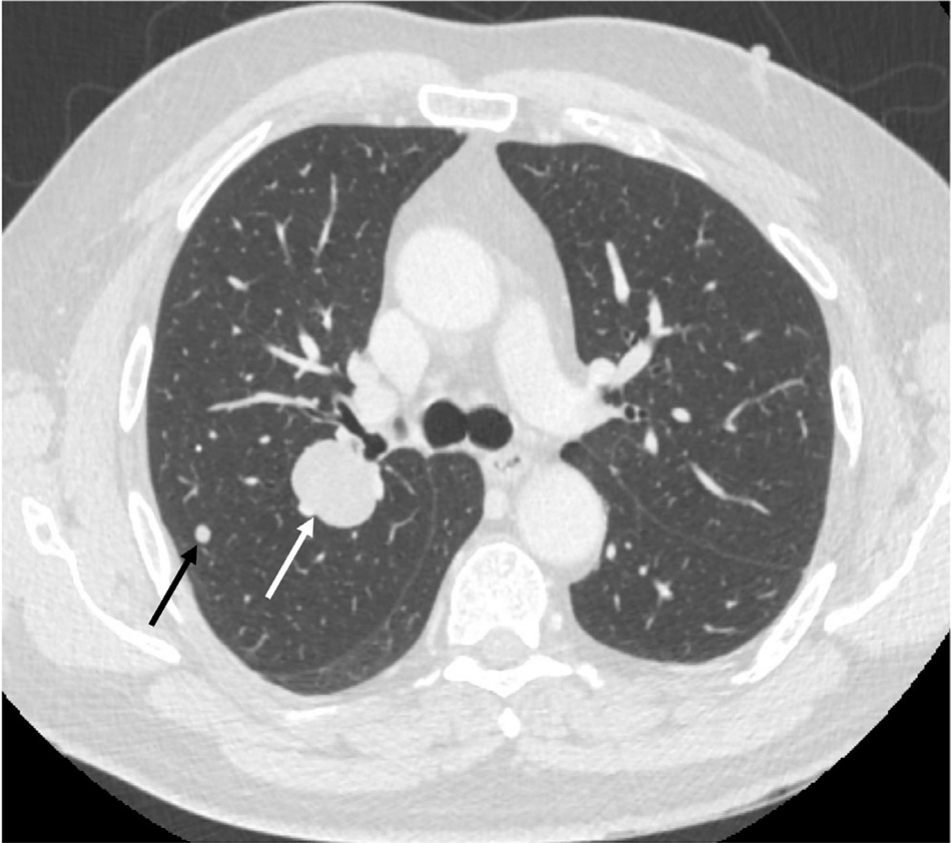
Selected thoracic CT axial slice (lung window, W:1500, L:-600) in a 58-year-old male presenting with a 3-month history of cough. The image demonstrates a 33 mm mass (white arrow) and a peripheral solid nodule in the posterior segment of the right upper lobe (black arrow). Multiple further bilateral pulmonary nodules were also identified (not shown).

A 58-year-old male presented to his general practitioner with a 3-month history of cough. An urgent chest radiograph demonstrated a suspicious mass in the right upper zone. A subsequent computed tomography (CT) scan of the thorax confirmed a 3 cm right upper lobe lung mass with multiple subcentimeter pulmonary nodules (Fig. 1). No other significant finding was identified on completion abdominopelvic CT.

**Fig. 2:**
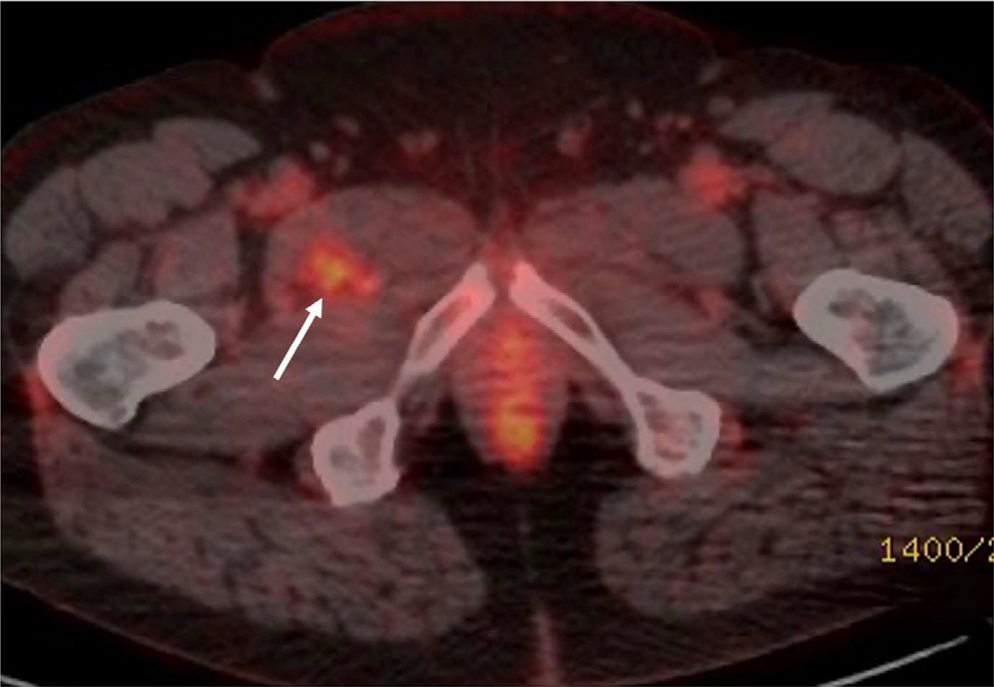
Selected PET-CT axial slice of the same patient demonstrates high FDG uptake in the right thigh anterior musculature (white arrow).

**Fig. 3:**
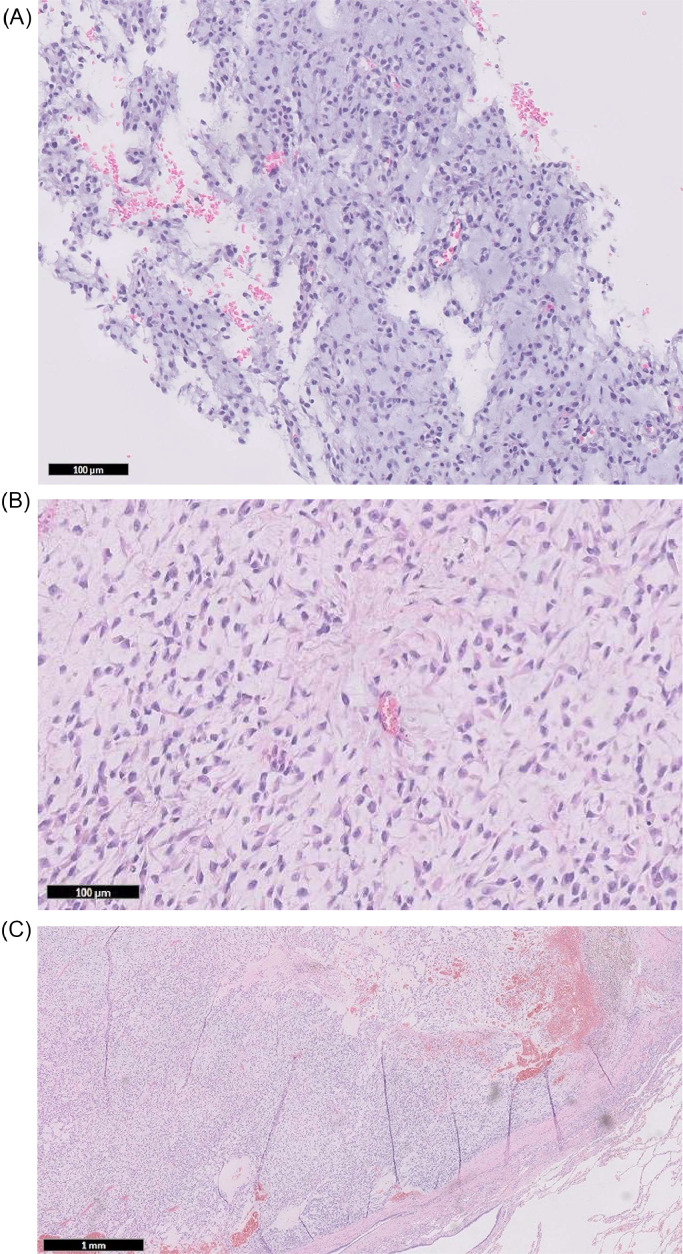

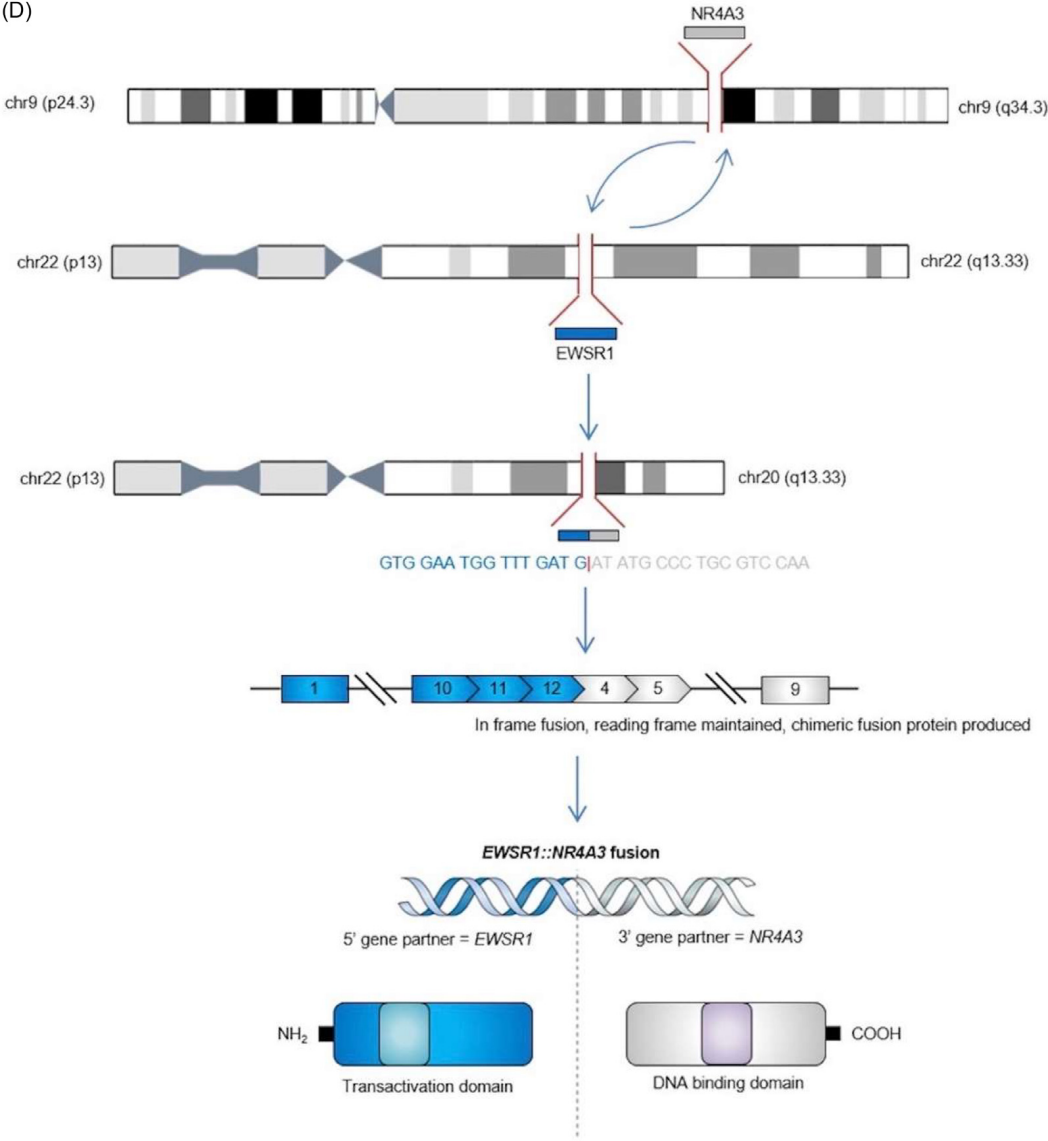
Selected histology slices and illustration. (A) H&E x40 (ultrasound-guided core biopsy of the right groin lesion). Myxoid matrix with the short epithelioid cells with a nuclear notch. (B) H&E x100 (ultrasound-guided core biopsy of the right groin lesion). The tumor consists of cords, nests and solid sheets of short ovoid to solid spindle cells with mild nuclear pleomorphism and single-cell apoptosis but no tumor necrosis. Mitotic activity is low (1 per 10 HPF). (C) H&E x10 (excised lung mass). Myxoid spindle cell tumor with myxoid stroma and a spindle cell population. There are chondroid foci present within the tumor. (D) Gene fusion schematic diagram of EWSR1::NR4A3 fusion.

A follow-up CT performed 4-months later demonstrated a marginal increase in the size of the lung mass but no lymphadenopathy. The subcentimeter pulmonary nodules remained unchanged. A positron emission tomography-computed tomography (PET-CT) examination demonstrated moderate fluorodeoxyglucose (FDG) tracer uptake within the right lung mass but also within the right groin, close to the right hip joint (Fig. 2). Histology from endobronchial ultrasound biopsy of the right upper lobe mass revealed mesenchymal spindle cell neoplasm with EWSR1 gene rearrangement. A working diagnosis of pulmonary myxoid sarcoma was considered; however, EMC was confirmed histologically following a right upper lobe wedge resection (Fig. 3).

**Fig. 4:**
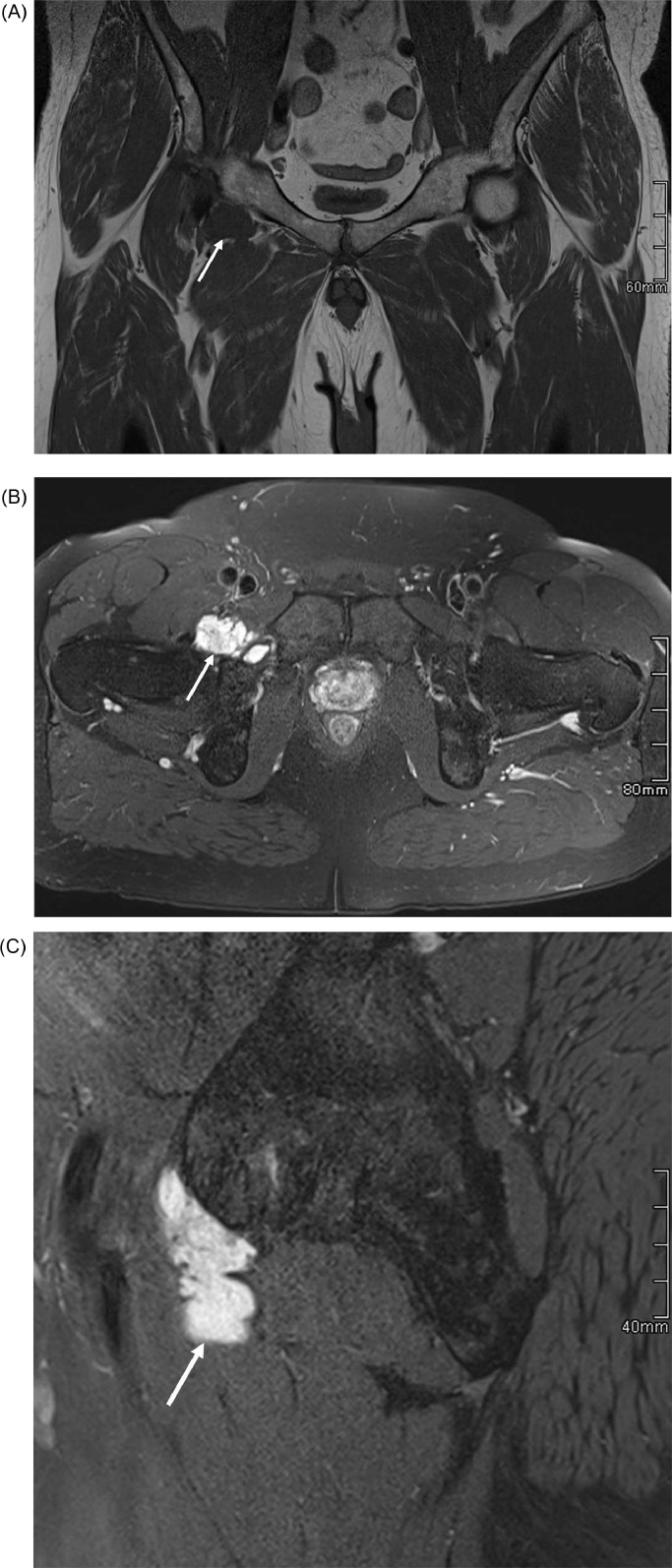
Selected pelvic MRI slices of the same patient. (A) Coronal T1 imaging demonstrates a homogenous low T1 signal lesion (white arrow) anterior to the right hip joint. (B) Axial proton density fat-saturated and (c) sagittal proton density fat-saturated slices demonstrate a lobulated high signal lesion (arrows) close to the hip joint extending into the area of the obturator externus bursa.

**Fig. 5:**
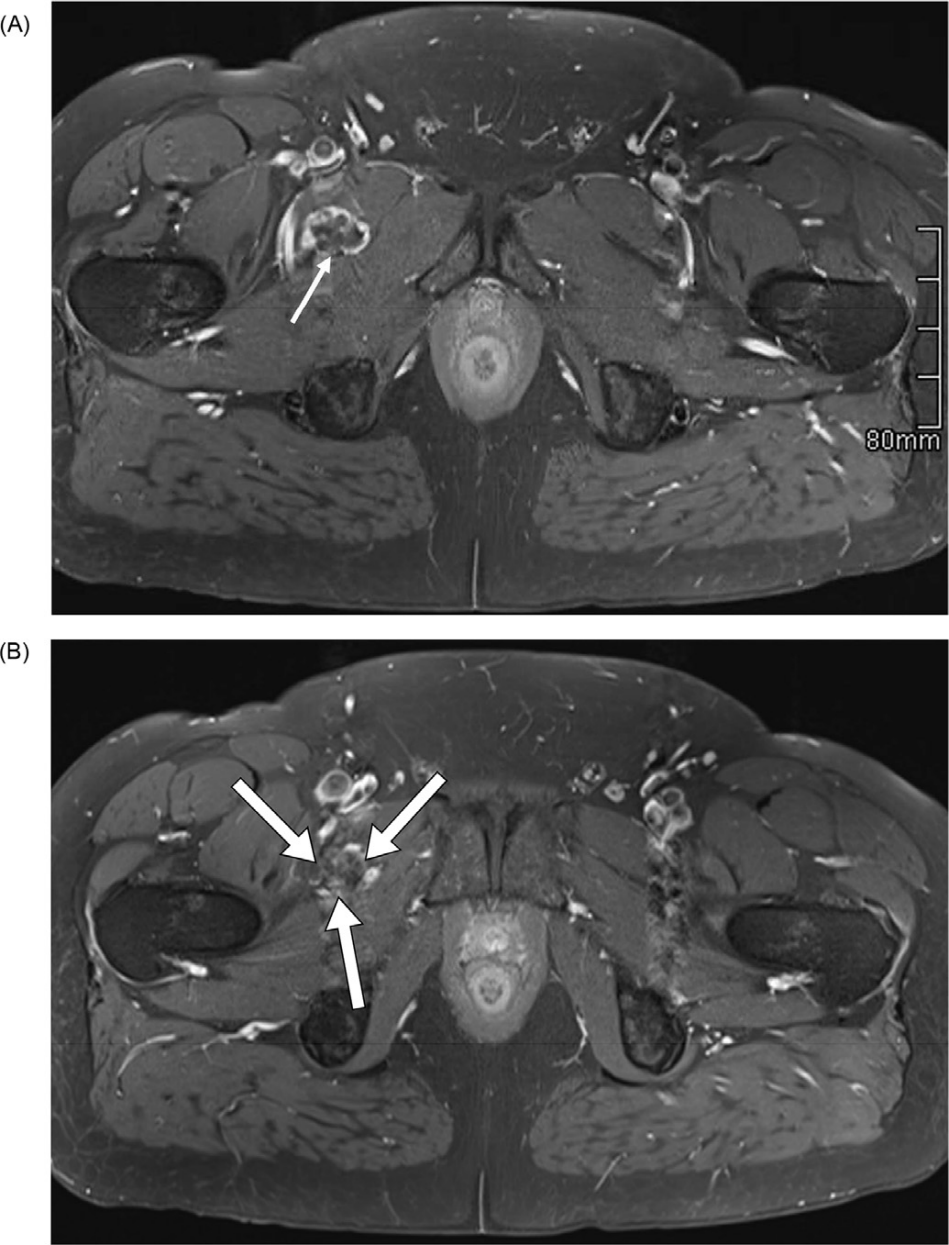
(A) and (B) Selected postcontrast fat-saturated T1 MRI pelvis axial slices of the same patient demonstrate a peripheral and septal enhancement pattern of the right groin lesion (arrows) with no central enhancement.

The patient was referred for a pelvic MRI given the right groin FDG uptake on PET-CT. This demonstrated a multi-lobulated lesion between the right pectineus and obturator externus muscles which was closely related to the anterior hip joint capsule. The lesion had a homogenous high signal on fluid-sensitive, fat-saturated sequences with internal septations and a homogenous intermediate signal on T1-weighted sequences (Fig. 4). A peripheral and septal enhancement pattern was noted following the administration of intravenous gadolinium (Fig. 5). The anterosuperior acetabular labrum contained a small, smooth cleft which did not communicate with the lesion. Appearances were felt most likely to represent a ganglion or paralabral cyst. A degenerative cyst was also present along the posterior articular surface of the femoral head with adjacent joint space narrowing and chondral loss. No other bone or soft tissue lesion was identified. The patient reported no symptoms relating to the right hip or groin. However, given the corresponding uptake within this lesion on PET-CT and the malignant histology of the lung mass, an ultrasound-guided biopsy of the right groin lesion was performed: subsequent histology was identical to that of the pulmonary nodules. As such, the right hip lesion was felt to represent the primary lesion with secondary metastatic pulmonary involvement.

A repeat CT scan performed 3-months after the groin biopsy demonstrated a mild increase in the size of the known pulmonary nodules consistent with metastases. Regarding the groin mass, the options of radiotherapy alone, debulking surgery followed by radiotherapy, or complete excision with potential consideration for complex hip reconstruction were discussed with the patient. He opted for surgical excision of the primary tumor with groin dissection and surgical removal of adjacent bone, including the superior pubic ramus and part of the anterior column. A histological clear resection margin was achieved following which the patient had an uneventful postoperative recovery.

Post-operative surveillance staging CT scans at 5-, 12- and 18 months demonstrated stable pulmonary disease and no local right groin recurrence. However, a repeat CT 29 months postsurgery demonstrated a mild increase in the size of the pulmonary metastases and a new 2 cm low attenuation lesion in the right groin adjacent to surgical clips consistent with local recurrence. The patient is being conservatively managed with regular clinical and imaging surveillance.

## Discussion

EMC, first described by Enzinger and Shiraki as a discrete entity in 1972 [1], is a rare malignant mesenchymal neoplasm of uncertain differentiation accounting for less than 3% of soft tissue sarcomas [2]. It has a male predilection and usually presents in the deep soft tissues of the proximal extremities and limb girdles (approximately 70%) with a subcutaneous location accounting for the remainder of cases (27%-33%) [3,4].

EMC more commonly involves the lower limbs. The upper extremities and the trunk are less affected [2]. In their study of 86 patients, Drilon et al. [5] reported that 62% of their patients presented with lower limb EMC. Kawaguchi et al. [3] reported lower limb involvement in 69% of cases in their study of 42 patients. Intra-articular lower limb EMC is rare with only a few cases reportedly involving the ankle [6,7], knee, and hip joints [8]. Brown et al. [10] found that 63% of cases occurred in the lower limb or around the hip in their study of 270 patients with EMC.

Presentation is most common in the sixth decade of life but EMC may also present in younger patients, although rarely in children [3,9–11]. It often has a prolonged and indolent clinical course with a high incidence of local recurrence and distant metastases [3,9,10]. Treatment involves wide local resection for local disease with or without radiotherapy [3,12].

EMC has a distinct histological morphology characterized by an abundant myxoid matrix with cells containing eosinophilic cytoplasm arranged in cords or clusters. Despite the name, there are no specific histological features which suggest chondrosarcoma [13]. Due to this myxoid matrix, EMC may mimic a multiseptated cystic lesion on MRI and a peripheral and septal enhancement pattern on MRI has been associated with the EWSR1::NR4A3 cytogenetic EMC subtype [4]. EMCs are defined by rearrangements of the NR4A3 gene. An EWSR1::NR4A3 fusion is predominantly noted in 75% of EMC cases with fewer cases having *NR4A3* fused to other gene partners including *TAF15, TCF12*, and *TFG*. In comparison to EWSR1::NR4A3 positive tumors, EMCs with variant *NR4A3* gene fusions demonstrate a: greater incidence of rhabdoid phenotype; high-grade morphology; and are associated with a more aggressive clinical course with poorer overall outcomes [14].

Well-recognized simple cystic lesions around the hip joint include ganglions, para-labral cysts, and bursae [8,15]. The most proximal portion of the lesion in our case was closely located to the anterior aspect of the hip joint capsule and labrum which extended into the region of the obturator externus bursa and obturator canal close to the obturator neurovascular bundle. The obturator externus bursa is a synovial lined space lying between the obturator externus tendon and the posteroinferior aspect of the hip joint capsule. There is the potential for this to communicate with the joint capsule and become distended in the presence of a hip joint effusion or synovial proliferation [16]. Moreover, several case reports have described ganglion or synovial cysts arising from this location resulting in obturator nerve entrapment [[17], [18], [19]–20]. The lesion in our patient was fully excised with clear pathological margins without breaching the hip joint capsule which confirmed that this lesion was external to the capsule and not intra-articular.

The MRI features of EMC were studied in a series of 12 patients by Tateishi et al*.* who reported that in most cases of EMC, lesions were typically multinodular masses demonstrating intermediate signal intensity on T1-weighted imaging with higher areas of T1 signal consistent with hemorrhage, and heterogenous high signal on T2-weighted imaging. There was a variable enhancement pattern within the solid components ranging from peripheral to diffuse or mixed enhancement. The peripheral enhancement pattern was seen more typically with the EWS:NR4A3 cytogenetic variant [4].

In their report of EMC presenting as an intra-articular ankle mass, Bhamra et al. [6] highlighted the need for intravenous gadolinium administration to differentiate the solid tumor component from a simple cystic lesion on MRI, the latter being recognized by the rim and septal contrast enhancement. In the case of our patient, the homogenous intermediate T1 signal, multiseptated high T2 signal and peripheral/septal enhancement pattern mimicked a large juxta-articular synovial cyst or ganglion with no central nodular enhancement to indicate a more solid tumor. The decision to proceed to biopsy was based on the moderate FDG uptake on PET-CT and the known metastatic lung disease, rather than the MRI features. Of note, the patient was completely asymptomatic with no reported symptoms from this right groin lesion.

Our case highlights that a peripheral and septal enhancement pattern, often demonstrated by EMC on MRI, has overlapping features with simple cystic lesions. Therefore, contrast enhancement is not a reliable discriminator in differentiating EMC from ganglion/synovial cysts as opposed to other tumors that demonstrate a more solid and diffuse enhancement pattern.

It should be recognized that EMC arising within or around a joint is rare. However, given that benign cystic lesions are commonly encountered in juxta-articular locations, these similarities may mislead radiologists and potentially delay definitive diagnosis and timely management.

## Patient consent

This case report was discussed with the patient and informed written consent was obtained.
