# Corrigendum to “Juxta-articular extraskeletal myxoid chondrosarcoma mistaken for a benign cyst presenting with multiple lung metastases” [Radiology Case Reports 19 (2024) 684-690]

**DOI:** 10.1016/j.radcr.2023.12.026

**Published:** 2024-01-03

**Authors:** Dmitriy Starostin, Ibrahim Azam, Michael Paddock, Malee S. Fernando, Scott Evans, Nikhil Kotnis

**Affiliations:** a Clinical Radiology Department, Sheffield Teaching Hospitals NHS Trust, Sheffield, UK; b Department of Radiology, Stoke Mandeville Hospital, Buckinghamshire Healthcare NHS Trust, Buckinghamshire, UK; c Medical Imaging Department, Perth Children's Hospital, Perth, Western Australia, Australia; d Division of Pediatrics, University of Western Australia, Perth, Western Australia, Australia; e School of Medicine, The University of Notre Dame Australia, Perth, Western Australia, Australia; f Division of Clinical Medicine, School of Medicine and Population Health, University of Sheffield, Sheffield, UK; g Sheffield Teaching Hospitals NHS Trust, Sheffield, UK; h The Royal Orthopedic Hospital, Birmingham, UK; i University of Birmingham, Birmingham, UK; j MSK Radiology Department, Sheffield Teaching Hospitals NHS Trust, Sheffield, UK

The authors would like to draw your attention to the following information, which was not included in their published article.

**Acknowledgments**

Funding was provided by the University of Sheffield Institutional Open Access Fund. For the purpose of open access, the author has applied a Creative Commons Attribution (CC BY) licence to any Author Accepted Manuscript version arising.

**Data availability**

All data supporting the findings of this study are available within the paper.

**Patient consent**

No.

